# Secretome characterization of clinical isolates from the *Mycobacterium abscessus* complex provides insight into antigenic differences

**DOI:** 10.1186/s12864-021-07670-7

**Published:** 2021-05-25

**Authors:** Fernanda Cornejo-Granados, Thomas A. Kohl, Flor Vásquez Sotomayor, Sönke Andres, Rogelio Hernández-Pando, Juan Manuel Hurtado-Ramirez, Christian Utpatel, Stefan Niemann, Florian P. Maurer, Adrian Ochoa-Leyva

**Affiliations:** 1 Departamento de Microbiología Molecular, Instituto de Biotecnología, Universidad Nacional Autonoma de México, Cuernavaca, Morelos Mexico; 2 Molecular and Experimental Mycobacteriology, Research Center Borstel, Borstel, Germany; 3 German Center for Infection Research (DZIF), Partner site Hamburg-Lübeck-Borstel, Borstel, Germany; 4 National and WHO Supranational Reference Center for Mycobacteria, Research Center Borstel, Leibniz Lung Center, Borstel, Germany; 5 Experimental Pathology Section, National Institute of Medical Sciences and Nutrition Salvador Zubirán, Mexico City, Mexico; 6 Institute of Medical Microbiology, Virology and Hospital Hygiene, University Medical Center Hamburg-Eppendorf, Hamburg, Germany

**Keywords:** Bioinformatics, Antigenicity, *M. abscessus* subspecies, In silico analysis, Vaccinology

## Abstract

**Background:**

*Mycobacterium abscessus* (MAB) is a widely disseminated pathogenic non-tuberculous mycobacterium (NTM). Like with the *M. tuberculosis* complex (MTBC), excreted / secreted (ES) proteins play an essential role for its virulence and survival inside the host. Here, we used a robust bioinformatics pipeline to predict the secretome of the *M. abscessus* ATCC 19977 reference strain and 15 clinical isolates belonging to all three MAB subspecies, *M. abscessus* subsp. *abscessus*, *M. abscessus* subsp. *bolletii*, and *M. abscessus* subsp. *massiliense*.

**Results:**

We found that ~ 18% of the proteins encoded in the MAB genomes were predicted as secreted and that the three MAB subspecies shared > 85% of the predicted secretomes. MAB isolates with a rough (R) colony morphotype showed larger predicted secretomes than isolates with a smooth (S) morphotype. Additionally, proteins exclusive to the secretomes of MAB R variants had higher antigenic densities than those exclusive to S variants, independent of the subspecies. For all investigated isolates, ES proteins had a significantly higher antigenic density than non-ES proteins. We identified 337 MAB ES proteins with homologues in previously investigated *M. tuberculosis* secretomes. Among these, 222 have previous experimental support of secretion, and some proteins showed homology with protein drug targets reported in the DrugBank database. The predicted MAB secretomes showed a higher abundance of proteins related to quorum-sensing and Mce domains as compared to MTBC indicating the importance of these pathways for MAB pathogenicity and virulence. Comparison of the predicted secretome of *M. abscessus* ATCC 19977 with the list of essential genes revealed that 99 secreted proteins corresponded to essential proteins required for in vitro growth.

**Conclusions:**

This study represents the first systematic prediction and in silico characterization of the MAB secretome. Our study demonstrates that bioinformatics strategies can help to broadly explore mycobacterial secretomes including those of clinical isolates and to tailor subsequent, complex and time-consuming experimental approaches accordingly. This approach can support systematic investigation exploring candidate proteins for new vaccines and diagnostic markers to distinguish between colonization and infection. All predicted secretomes were deposited in the Secret-AAR web-server (http://microbiomics.ibt.unam.mx/tools/aar/index.php).

**Supplementary Information:**

The online version contains supplementary material available at 10.1186/s12864-021-07670-7.

## Background

Non-tuberculous mycobacteria (NTM) are widely disseminated, mostly saprophytic and partly opportunistic bacteria. The prevalence of NTM in clinical specimens has increased globally, and in some industrialized countries, infections caused by NTM are becoming more common than tuberculosis (TB). Infections caused by *M. abscessus* (MAB) are particularly challenging to manage due to the extensive innate resistance of MAB against a wide spectrum of clinically available antimicrobials [1]. MAB causes mostly pulmonary and occasionally extrapulmonary infections that can affect all organs in the human body [2]. Current treatments for MAB induced pulmonary disease are long, associated with severe side effects and a cure rate below 50% [3–5]. MAB is comprised of three subspecies, *M. abscessus* subsp. *abscessus, M. abscessus* subsp. *bolletii* and *M. abscessus* subsp. *massiliense*, hereafter referred to as MAB_A,_ MAB_B_, and MAB_M_, respectively [6]. MAB isolates can show smooth (S) and rough (R) colony morphotypes, a trait that relies on the presence (S) or absence (R) of surface-associated glycopeptidolipids (GPLs) and that correlates with the virulence of the strain [7–10]. Transitioning from high-GPL to low-GPL production is observed in sequential MAB isolates obtained from patients with chronic underlying pulmonary disease. In these patients, S-to-R conversion is thought to present a selective advantage as the aggregative properties of MAB R variants strongly affect intracellular survival. The selective advantage is also related to the loss of immunogenic GPLs. In addition, a propensity to grow as extracellular cords allows these low-GPL producing bacilli to escape innate immune defenses [10].

The complete set of proteins excreted / secreted (ES) by a bacterial cell is referred to as its secretome. The secretome is involved in critical biological processes such as cell adhesion, migration, cell-to-cell communication and signal transduction [11] ES proteins are considered an important source of molecules for serological diagnosis. Also, secreted proteins can be highly antigenic due to their immediate availability to the host immune system and are thus of interest in vaccinology [12, 13]. So far, there have been few efforts to experimentally determine the secretome of MAB, and in particular, the secretomes of clinical MAB isolates [14–17]. Nowadays, sequencing and bioinformatics strategies can be explored for the systematized prediction of ES proteins from bacterial genomes [18, 19]. Recently, a robust bioinformatics pipeline for predicting and analyzing the complete in silico secretome of two clinical *M. tuberculosis* (MTB) genomes was published showing higher overall agreement with an experimental secretome compiled from literature than two previously reported secretomes for *M. tuberculosis* H37Rv [19].

To gain further insights into MAB ES proteins and their association with virulence and pathogenicity we here sequenced and assembled the genomes of 15 clinical MAB isolates belonging to all three subspecies including S and R morphotypes. We then adapted the bioinformatics strategy previously established for MTB to predict and analyze the complete set of ES proteins encoded in these isolates and in the *M. abscessus* ATCC 19977 type strain, and compared it with our previous findings for MTB [19].

## Results

### Genome assembly, secretome prediction and annotation

We sequenced the genomes of 15 pulmonary and extrapulmonary (skin, tissue, lymph node, and blood) MAB isolates *obtained* from patients in Germany comprising all three MAB subspecies (Table 1 and Additional file 1: Table S1)*.* For each genome, we obtained an average of 2,601,444 quality-filtered reads. After de novo assembly, we obtained from 38 to 78 contigs (mean = 58 contigs) with genome coverage of 217- to 368-fold (mean = 310-fold) and with an average of 5082 total proteins per genome (Additional file 3: Table S2). Also, we performed a Multilocus Sequence Typing (MLST) analysis at the Pasteur Institute site (https://bigsdb.pasteur.fr/cgi-bin/bigsdb/bigsdb.pl?db=pubmlst_mycoabscessus_seqdef) to assess the genetic variability among the studied samples. This analysis assigns a Sequence Type (ST) to each strain by looking for sequence variations in seven housekeeping genes and providing information about philogenetic relationship [20]. We observed that eight out of 15 genomes had unique STs, three genomes were not defined and notably, two genomes belonged to ST 117 while other two belonged to ST 52, suggesting they could be highly related (Table 1).

**Table 1:** Clinical isolates metadata and number of ES proteins

Strain	Accession number	Genome ID	Origin	Phenotype	Sequence Type (ST)	Total predicted proteins	ES proteins	% ES proteins
*M. abscessus subsp. abscessus*	GCA_015499845.1	4549-15	sputum	rough	1	5105	929	18
	GCA_015499865.1	11351-15	sputum	rough	63	5138	966	19
	GCA_015499835.1	8844-15	skin	smooth	246	4854	956	20
	GCA_015499805.1	3563-15	sputum	smooth	33	5239	968	18
	GCA_015499795.1	12389-15	sputum	smooth	47	5276	990	19
	GCA_015499765.1	2677-16	sputum	smooth	34	4900	919	19
	GCA_015499745.1	2572-17	tissue (breast implant)	NA	10-46-64-70-261	4847	874	18
*M. abscessus subsp. massiliense*	GCA_015499715.1	14479-15	sputum	rough	117	5120	962	19
	GCA_015499735.1	10896-16	sputum	rough	117	5109	950	19
	GCA_015499695.1	10003-15	sputum	smooth	98-245-271	4835	891	18
	GCA_015499655.1	16155-15	sputum	smooth	98-245-271	4884	898	18
*M. abscessus subsp. bolletii*	GCA_015499665.1	11702-16	sputum	rough	161	5079	931	18
	GCA_015499625.1	713-16	lymph node	rough	52	5456	1037	19
	GCA_015499615.1	7742-15	blood culture	smooth	333	4913	885	18
	GCA_015499585.1	13116-16	lymph node	smooth	52	5305	990	19
*M. abscessus subsp. abscessus*	CU458896.1	reference strain ATCC19977	–	–		4942	886	18
*M. tuberculosis H37Rv*	NC_000962.3	reference strain	–	–		4337	548	13

We used a bioinformatics pipeline previously reported by our group [19] to predict the full secretome of all MAB clinical isolates and the widely used reference strain *M. abscessus* ATCC 19977 (GenBank CU458896.1) (Additional file 2: Fig. S1). We obtained an average of 939 ES proteins per genome, representing ~ 18% of the total proteome (Table 1). The predicted secretome for the MAB reference strain consisted of 886 proteins. All these proteins showed a BLASTP hit against the NR database but only 494 (55.8%) could be annotated with GO terms.

**Fig. 1:**
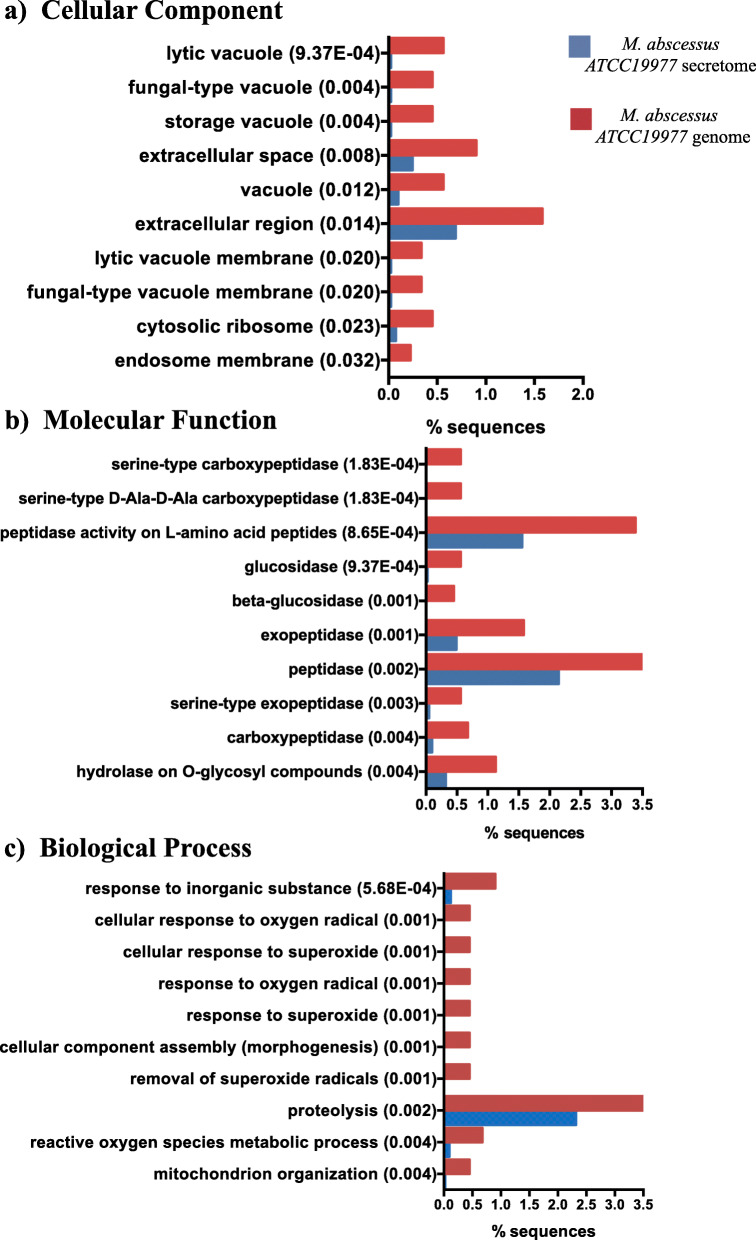
GO enrichment analysis for the *M. abscessus* ATCC 19977 reference strain. Top 10 most enriched GO terms for the *M. abscessus* ATCC 19977 secretome (blue) and complete genome (red) in three categories: **a** Cellular Component, **b** Molecular Function and **c** Biological Process

We analyzed the over-representation of GO terms in the secretome of *M. abscessus* ATCC 19977 as compared to the whole genome. The most significantly enriched GO-terms were: “lytic vacuole” (*p* = 9.37E-04), and “fungal-type vacuole” (*p* = 0.004) in Cellular Component (Fig. 1a), “serine-type carboxypeptidase” (*p* = 1.83E-04), and “serine-type D-Ala-D-Ala carboxypeptidase” (*p* = 1.83E-04) activities in Molecular Function (Fig. 1b) and, “response to inorganic substance” (*p* = 5.68E-04) and “cellular response to oxygen radical” (*p* = 0.001) in the Biological Process category (Fig. 1c). The KEGG pathway mapping of the ES proteins showed that 214 proteins (24.2%) could be assigned to 100 different KEGG pathways (Table 2), with the ABC transporter pathway being the most abundant (*n* = 13, 1.47%). Additionally, serine-type D-Ala-D-Ala carboxypeptidases (*p* = 1.83E-04) and peptidases (*p* = 8.40E-04) were the most significantly abundant enzymes according to the Enzyme Commission (EC) Classes (Additional file 6: Fig. S2), while the Mce/MiaD and PknH-like extracellular domains were the most enriched protein domains (Table 3). Of note, comparably few sequences were assigned to the PE/PPE category (*n* = 3). Notably, after comparing the predicted secretome of *M. abscessus* ATCC 19977 with a list of essential genes published by Laencina et al. [17], we found that 99 (11.17%) of the predicted ES proteins, corresponded to essential proteins required for in vitro growth.

**Table 2:** Top 10 KEGG pathways assigned for *M. abscessus* ATCC19977 ES proteins

Ranking	Pathway name	Number of represented ES proteins (%)
1	ABC transporters	13 (1.47)
2	Two-component system	9 (1.02)
3	Quorum sensing	6 (0.68)
4	Oxidative phosphorylation	4 (0.45)
5	Sulfur metabolism	4 (0.45)
6	Glycerolipid metabolism	4 (0.45)
7	Peptidoglycan biosynthesis	4 (0.45)
8	Protein export	4 (0.45)
9	Starch and sucrose metabolism	3 (0.34)
10	Glyoxylate and dicarboxylate metabolism	3 (0.34)

**Table 3:** Top 10 most represented protein domains in *M. abscessus* ATCC19977 secretome

InterProcode	InterPro description	Number of ES proteins (%)
IPR003399	Mce/MlaD	19 (2.14)
IPR026954	PknH-like extracellular domain	15 (1.69)
IPR032407	Haemophore, haem-binding	10 (1.13)
IPR020846	Major facilitator superfamily domain	7 (0.79)
IPR013766	Thioredoxin domain	6 (0.68)
IPR000064	Endopeptidase, NLPC/P60 domain	6 (0.68)
IPR001638	Solute-binding protein family 3/N-terminal domain of MltF	6 (0.68)
IPR000675	Cutinase/acetylxylan esterase	6 (0.68)
IPR005490	L,D-transpeptidase catalytic domain	5 (0.56)
IPR000073	Alpha/beta hydrolase fold-1	5 (0.56)

### Comparison of *M. abscessus* subspecies core secretomes

**Fig. 2:**
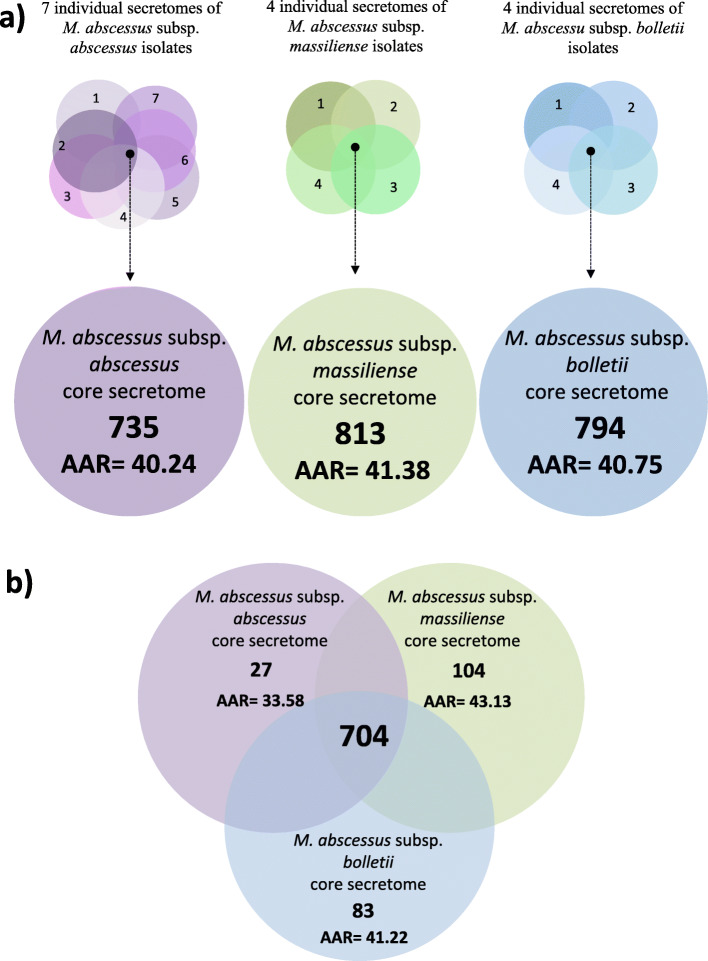
Venn diagram between the core secretomes of the three *M. abscessus subspecies*. **a** Number of total proteins contained in the core secretome of each subspecies. **b** Shared and unique proteins between the three subspecies as per BLASTP (E-value 1.0E-3)

We analyzed the differences between the predicted secretomes of the three MAB subspecies. To this end, we defined the core secretome of each subspecies as the set of proteins shared between all secretomes of isolates belonging to MAB_A_, MAB_B_, and MAB_M_, respectively. The resulting core secretomes contained 735 (MAB_A_), 794 (MAB_B_), and 813 (MAB_M_) proteins (Fig. 2a).

Given that our study considered a limited number of de novo assembled genomes, we additionally compared the predicted core secretomes to 60 additional MAB genomes available in NCBI (Additional file 4: Table S3). We found that an average of 99.78, 99.12, and 98.59% of our core secretomes was also present in the additional MAB_A_, MAB_B_, and MAB_M_ genomes, respectively, further corroborating the validity of the predicted subspecies core secretomes for other MAB isolates.

We then determined the respective Abundance of Antigenic Regions (AAR) values to estimate antigenic densities for the protein sets in each core secretome. The average AAR values from most to least antigenic were: 40.24 for MAB_A_, 40.75 for MAB_B_, and 41.38 for MAB_M_ with no statistically significant difference between them.

Next, we identified the ES proteins shared between the MAB_A_, MAB_B_, and MAB_M_ core secretomes. We found that 704 proteins (86.5%) were shared among MAB_A_, MAB_B_, and MAB_M_ with an AAR value of 41.17 (Fig. 2b). The AAR values for the protein sets exclusively found in the MAB_A_, MAB_B_, or MAB_M_ secretome were 33.58, 41.22, and 43.13, respectively, with the MAB_A_ dataset showing a significantly lower AAR value indicating higher antigenicity than the others (*p* < 0.1; Fig. 2b).

### Differences in core secretomes between R and S morphotypes

**Fig. 3:**
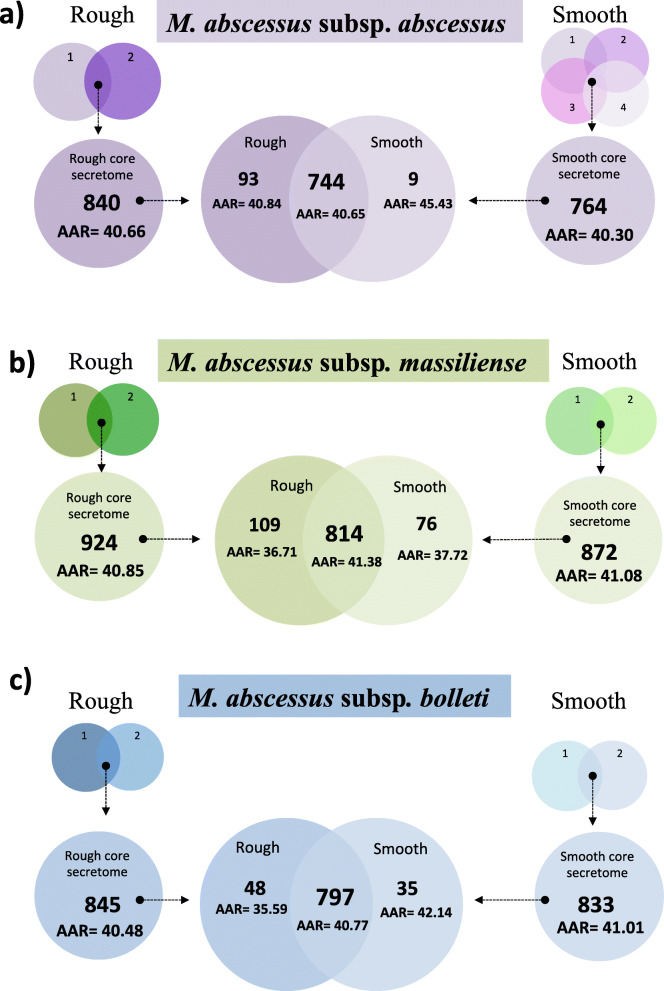
Venn diagram between the core secretomes of the three *M. abscessus* subspecies by colony morphotype. We used BLASTP (E-value 1.0E-3) to assess the core secretomes for isolates with rough and smooth colony morphotypes **a** *M. abscessus* subsp. *abscessus*, **b** *M. abscessus* subsp. *massiliense* and **c** *M. abscessus* subsp. *bolletii*

As MAB isolates with R and S morphotypes show differences in virulence and pathogenicity, we compared the predicted core secretomes of R and S isolates (Fig. 3). We observed that the core secretomes of R variants were larger (840, 924 and 845 proteins for MAB_A_, MAB_M_, and MAB_B_) than those of the investigated S variants (764, 872 and 833 proteins, respectively) with no significant differences in antigenic densities as per mean AAR value (Fig. 3). Intra-subspecies comparison of S and R secretomes revealed that 96.4, 90.7 and 95% of the identified ES proteins were found in both R and S morphotypes for MAB_A_, MAB_M_ and MAB_B_ respectively. The number of unique proteins was larger in the core secretome of the R morphotypes (*n* = 93, 109, and 48 for MAB_A_, MAB_M_, and MAB_B_) as compared to the S morphotypes (*n* = 9, 76, and 35, respectively; Fig. 3).

Interestingly, antigenic densities for the unique ES proteins of the R morphotypes were higher (AAR = 40.84, 36.71, and 35.59 for MAB_A_, MAB_M_, and MAB_B_) than for the proteins exclusive to the S morphotypes irrespective of the subspecies (AAR = 45.43, 37.72, and 42.14; Fig. 3). To assess if the AAR values of these specific protein sets were different from same-sized protein sets randomly chosen from the respective core secretomes, we created 1000 random sets of 109, 93, 76, 48, 35 and 9 proteins and calculated the AAR value for each set. Then, we determined an empirical *p*-value based on the number of random protein sets that equaled or exceeded the AAR value for each protein dataset as was previously suggested by Cornejo-Granados et al. [19]. We found that the ES proteins exclusive to the R morphotypes of MAB_M_ and MAB_B_ had significantly (*p* < 0.05) higher antigenic densities than randomly constructed protein sets (Additional file 5: Table S4).

Finally, we determined the MAB core secretomes by sample origin (pulmonary, extrapulmonary, blood). This resulted in 706 ES proteins shared among the ten pulmonary isolates, 758 proteins shared among the four extrapulmonary isolates, and 885 proteins for the single isolate grown from a blood sample. However, as per the GO, KEGG, and antigenicity analyses, we did not find any distinct characteristics specific to either sample source and, hence, type of infection.

### Antigenicity of ES and non-ES proteins

**Fig. 4:**
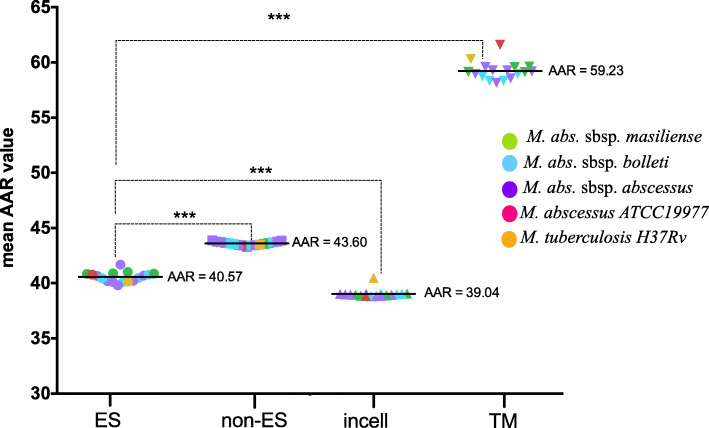
Comparison between AAR values for Excreted/Secreted (ES), non Excreted/Secreted (non-ES), intracellular (incell) and transmembrane (TM) proteins. AAR values were calculated for each of the 15 genomes sequenced. The X-axis shows the cellular compartment and the Y-axis shows AAR values for the genomes of each subspecies: *M. abscessus* subsp. *abscessus* (green), *M. abscessus* subsp. *bolletii* (blue), *M. abscessus* subsp. *massiliense* (purple), *M. abscessus* ATCC19977 (red) and *M. tuberculosis* H37Rv (orange). Mann-Whitney tests were performed to compare the AAR of each group with a confidence level of 99% (***, *p* < 0.001)

It has previously been reported for different microorganisms including MTB that ES proteins tend to be more antigenic than non-ES proteins [18, 19, 21]. We thus tested if this was also true for the investigated MAB isolates. First, we found that the antigenic densities as indicated by mean AAR values were similar among all isolates irrespective of subspecies or morphotype within the same cell compartment, i.e. for ES, non-ES, intracellular (incell) and transmembrane (TM) proteins (Fig. 4). Second, we found that antigenic densities were significantly higher in ES proteins as compared to non-ES proteins in all isolates (AAR = 40.57 and 43.60, respectively; *p*-value < 0.0001) (Fig. 4). However, within the non-ES category, incell proteins showed even higher antigenic densities (AAR = 39.04) than the predicted ES proteins (*p* < 0.0001) while the lowest overall antigenic densities were observed for the TM category (AAR = 59.23; *p* < 0.0001).

### Comparison of *M. abscessus* and *M. tuberculosis* secretomes

**Fig. 5:**
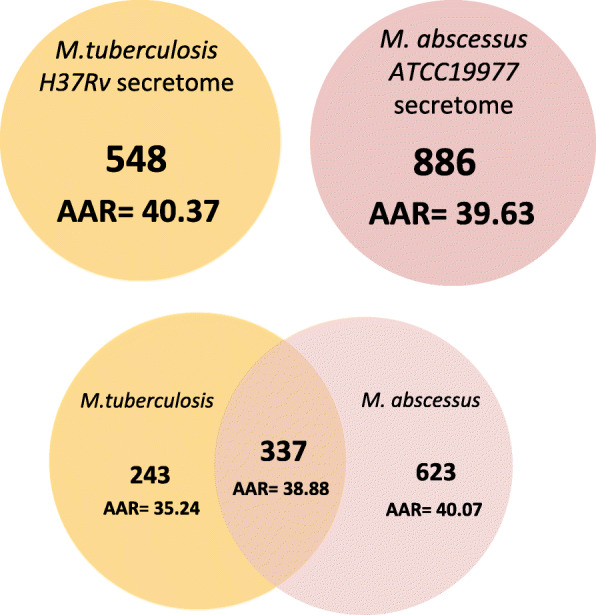
Venn diagram between the predicted secretomes of *M. tuberculosis* H37Rv and *M. abscessus* ATCC 19977. We used BLASTP (E-value 1.0E-3) to compare the complete secretomes of both species

Lastly, we compared the predicted secretome of *M. abscessus* ATCC 19977 against the previously reported secretome of *M. tuberculosis* H37Rv [19]. We observed that the *M. abscessus* secretome was predicted to be almost equally antigenic (AAR = 39.63) than the *M. tuberculosis* secretome (AAR = 40.37) (Fig. 5). We found 337 MAB ES proteins (38.04%) with homology to proteins in the predicted MTB secretome (Fig. 5). Interestingly, 222 of these proteins had sequence homology with proteins experimentally reported as secreted in MTB (comparable experimental secretome data for MAB was not available to us) [19] (Additional file 7: Table S5). Furthermore, we determined the average AAR value of the 680 ES proteins shared among the 15 MAB isolates (AAR = 41.53). This value means that antigenic density was lower than for the predicted secretome of *M. tuberculosis* H37Rv (AAR = 40.37) and two clinical *M. tuberculosis* isolates belonging to the Beijing lineage (isolate C3 AAR = 37.52 and isolate C4 AAR = 37.55) (Table 4) [19]. Finally, we identified 13 ES proteins with homologues in both MAB and *M. tuberculosis*, which are listed as targets for various FDA approved drugs (Additional file 8: Table S6).

**Table 4:** Abundance of Antigenic Regions (AAR) for *M. abscessus* and *M. tuberculosis* strains

Strain	Number of proteins in the dataset	Average AAR value
*M. tuberculosis* Beijing isolate C3^a^	553	37.52
*M. tuberculosis* Beijing isolate C4^a^	519	37.55
*M. bovis* BCG Pasteur	564	38.99
***M. abscessus*** **ATCC 19977**	**886**	**40.78**
*M. tuberculosis* H37Rv	548	40.37
*M. abscessus* clinical isolates	680	41.54

^a^ Both Beijing isolates were previously reported in Cornejo-Granados et al. [19]

## Discussion

This is the first study that proposes a method for prediction of MAB secretomes based on 15 clinical MAB isolates and the *M. abscessus* ATCC 19977 reference strain. Our results show that an average of 18% (939 proteins) of the total proteins encoded in the MAB core genome carry sequence patterns indicative of secretion. Notably, this percentage is 6% greater than the proportion previously reported for several MTB isolates (~ 12%) [19]. Nearly 200 species of mycobacteria have been identified with diverse lifestyles and a high degree of morphological, biochemical, and physiological diversity and a comparative genome analysis suggests that only a relatively small number of genes (1080) are shared between several *Mycobacterium* species [22, 23]. Moreover, loss of ancestral genes is a well described phenomenon in slowly growing mycobacteria such as MTB and, in particular, *M. leprae* [24]. In contrast, rapidly growing NTM such as MAB are considered to represent a more ancient evolutionary state, with larger genomes than those of MTB [23, 24]. Thus, it is not surprising that we found a larger number of ES proteins in MAB than MTB. Furthermore, the increased abundance of ES proteins in MAB as compared to MTB could be related to the ability of MAB to cause a different spectrum of disease and to adapt to different environmental settings requiring frequent interaction with a wide variety of host cells and organisms competing for the same ecological niche, likely involving cross species exchange of genetic information, for example by plasmid transfer [25–27]. A similar hypothesis has been suggested for fungal secretomes [28].

The GO and KEGG pathway annotations of the secretomes of *M. abscessus* ATCC 19977 and the MAB clinical isolates showed enrichment consistent with the characterization of previously reported mycobacterial secretomes [18, 19]. Interestingly and in line with the increased secretome size as compared to MTB, the KEGG pathway analysis showed a high abundance of the Quorum sensing pathway for the predicted MAB secretomes, which was not present in our previous MTB secretome pathway analysis [19]. The presence of a Quorum sensing pathway would be another similarity shared between MAB and non-mycobacterial pathogens commonly affecting patients with chronic lung disease such as *Pseudomonas aeruginosa* [29]. In addition, it could be related to the ability of MAB to form biofilms [30, 31], further contributing to the capacity of MAB to tolerate antibiotics and to persist over long periods in the environment [32–35].

The InterPro annotation showed that Mce domains were the most abundant (2.14%) domains in the MAB reference secretome, while PPE and PE-PGRS domains only corresponded to 0.3% of the ES protein sequences. This tendency is contrary to our observations for MTB [19], where the PPE and PE-PGRS domains accounted for ~ 12% of the secreted proteins and the Mce domains for only 0.5%. The lower quantity of predicted PE/PPE proteins in MAB was somewhat expected. *M. tuberculosis* has five ESX secretion systems, four of which encode PE/PPE proteins, while MAB has only two (ESX-3 and ESX-4) of which only the ESX-3 operon includes PE/PPE genes [36]. In contrast, Mce domains are known for participating in host cell entry by mycobacteria [37]. Thus, their higher abundance in MAB as compared to MTB highlights the importance of this pathway for MAB survival within the host. It needs to be mentioned though that Kumar et al. [37], also suggested that in low virulence bacteria, transport activities could be the primary function of Mce operons.

To compare the predicted secretomes according to colony morphotype, we first established the core secretome for the R and S variants per subspecies, thus eliminating individualities among the different isolates (Fig. 3). The high overall agreement between the core secretomes for both morphotypes of approximately 90% was expected, considering the fact that R variants can arise from the S morphotypes during persistent infection by loss of surface-exposed GPLs caused by mutations in the GPL synthesis pathway [26, 38–40]. However, both the higher number and the higher antigenic densities (lower AAR values) of the ES proteins exclusively found in R variants indicate that additional genetic changes may evolve during S-to-R conversion. Moreover, this observation raises the question whether some strains with additional genetic traits associated with virulence are able to undergo S-to-R conversion and cause disease due to R variants more easily than others. Genomic studies involving sequentially isolated S and R variants of the same strain obtained from individual patients over time will be required to better characterize the microevolution of MAB strains within the chronically infected host.

Similarly, the fact that MAB causes both chronic pulmonary disease (with R variants sometimes increasing over time) and extrapulmonary manifestations (mostly caused by S variants) led us to investigate whether differences exist in the predicted secretomes of isolates related to these clinical presentations. The absence of major differences in the GO, KEGG, and antigenicity analyses suggest that secretome variations do not influence MAB tissue tropism. Consequently, host characteristics such as severe immunosuppression may be the main driver for invasive MAB infections. Likewise, in the case of tissue infections, which often occur following surgical interventions, insufficient hygiene procedures and sterilization protocols for surgical equipment appear to be more relevant than pathobiological traits such as the secretome intrinsic to the causative MAB isolate [41].

Lastly, we observed that the predicted secretomes of all investigated clinical MAB isolates were less antigenic than the secretomes of *M. tuberculosis* H37Rv and two clinical *M. tuberculosis* isolates. Additionally, although there was no statistical difference, the isolates with a rough phenotype tended to be more antigenic than the isolates with a smooth phenotype. Previous evidence with *M. tuberculosis* [19] showed that clinical isolates from the Beijing phenotype showed increased virulence and less antigenic secretomes than the reference strain H37Rv. Thus, the diminished antigenicity of MAB could be viewed as a virulence trait in itself as it would support colonization of the host for extended time periods without immediate progression into clinical disease. However, further experimental tests on antigenicity are needed to demonstrate this observation.

This study represents the first systematic prediction and in silico characterization of the MAB secretome. We acknowledge that an important constraint in this study is the limited total number of genomes analyzed per subspecies and biological source. Thus, care must be taken to not over interpret the findings related to sample subcategories such as subspecies and morphotypes. Also, published experimental data on MAB secretomes are very limited and no systematic validation of the in silico findings reported herein could be performed against such datasets. Although more research will be needed to determine experimental secretomes in NTM, our study demonstrates that using bioinformatics strategies can help to broadly explore mycobacterial secretomes including those of clinical isolates and to tailor subsequent, complex and time-consuming experimental approaches accordingly. This approach can support a systematic investigation of mycobacterial secretomes exploring candidate proteins suitable for developing new vaccines and diagnostic markers to distinguish between colonization and infection.

## Methods

### Clinical isolates

We selected 15 MAB clinical isolates comprising members of all MAB subspecies (MAB_A_, *n* = 7; MAB_B_, *n* = 4*;* MAB_M_, n = 4) and both S (*n* = 8) and R (*n* = 6) morphotypes (not determined, *n* = 1). The strains were isolated from different biological sources representing both pulmonary colonization / infection (sputum, *n* = 10) and extrapulmonary samples (skin, *n* = 1; soft tissue, *n* = 1; lymph nodes, *n* = 2; blood, *n* = 1) (Table 1 and Additional file 1: Table S1). For routine diagnostic purposes, species identification was performed using GenoType NTM-DR line probe assays (HAIN Lifescience, Nehren, Germany) and sequencing of the 16S and *rpoB* genes as described previously [42].

### Whole genome sequencing and genome assemblies

Genomic DNA (gDNA) of the 15 MAB clinical isolates was extracted from solid cultures using a Centrimonium bromide chloroform DNA extraction protocol as previously described [43]. DNA libraries were constructed with the Nextera XT kit from Illumina and sequenced on the Illumina MiSeq benchtop platform with a v3 chemistry paired –end run and a read lenght of 2 × 300 bp. We processed the resulting reads with Trimmomatic [44], clipping the Illumina adapter sequences and trimming the reads with a sliding window of 20 bp looking for quality > 30 and discarding all reads shorter than 100 bp. Trimmed reads were used to construct de novo assemblies using SPADES [45] with default parameters and the --careful option enabled. Then, each assembly was analyzed with RAST [46] to obtain all the open reading frames (ORFs). Additionally, we predicted the ORFs from the deposited genome sequence of the *M. abscessus* ATCC 19977 type strain (GenBank CU458896.1) (Additional file 1: Table S1).

### Secretome prediction

The complete set of predicted ORFs was independently analyzed for each genome using the bioinformatics pipeline previously reported by Cornejo-Granados et al. [19] and summarized in Additional file 2: Figure S1. Briefly, we used six different feature-based tools (SignalP, SecretomeP, LipoP, TatP, TMHMM and Phobius) [47–51] to identify ES proteins by the different secretion pathways and to remove the ones that had transmembrane domains (Additional file 2: Fig. S1). The proteins assigned as not-secreted (non-ES) were further classified into transmembrane proteins (TM) if they showed the presence of transmembrane domains with TMHMM 2.0 [50], and into intracellular proteins (incell) if they did not contain any transmembrane domains.

### Annotation and comparative analysis of secreted proteins

To assign functional annotations to the proteins present in our genomes, we performed a BLASTP query of those proteins against the non-redundant (nr) complete database using Blast2GO [52] with an E-value cut-off set at 1.0E-3. Furthermore, all proteins were associated with protein families through InterProScan [53] and functionally mapped to Gene Ontology (GO) terms by setting the following parameters: E-value-hi-filter: 1.0E-3; Annotation cut-off: 55; GO weight: 5 and Hsp-Hit Coverage cut-off: 0. Blast2GO was then used to identify over- and under-represented GO and Enzyme Commission (EC) numbers in the ES proteins by setting the significance filter *p*-value to ≤0.05. Also, we used the KEGG Automatic Annotation Server (KAAS) database [54] to assign the pathway annotation to the secreted proteins using the BBH (bidirectional best hit) method and the reference gene data set assigned to *Mycobacterium*.

To determine differences between the predicted secretomes in relation to MAB subspecies and morphotype, we established core secretomes by performing a bidirectional best-hit BLASTP search (E-value 1.0E− 3) between the ES proteins of all genomes belonging to the respective subspecies and morphotypes. Then, we identified the shared and unique proteins for each comparison. Additionally, we determined the set of homologous ES proteins shared between the MAB reference strain ATCC 19977 and both *M. tuberculosis* H37Rv predicted and experimental secretomes [19]. The resulting proteins were further investigated for sequence similarities against known drug targets available on the Drug Bank database (http://www.drugbank.ca/), setting the E-value to 1.0E-3 and all other options to default. In Additional file 8: Table S6, we show all proteins that have similarity with an approved drug target, as well as the drugs that can affect said target.

Additionally, we analyzed the presence of the core secretomes in 20 *M. abscessus* genomes per subspecies downloaded from NCBI (Additional file 4: Table S3). To this end, each downloaded genome was analyzed with RAST to obtain all the open reading frames (ORFs). Next, we performed a BLASTP search (E-value 1.0E− 3) of each core secretome against each genome of the corresponding subspecies, and all hit proteins were considered homologs.

### Calculation of the abundance of antigenic regions

The Abundance of Antigenic Regions (AAR) value is used to estimate the antigenic density of a protein by calculating the number of antigenic regions and normalizing it to the sequence length [18]. Of note, proteins with higher antigenic densities have lower AAR values. For this study, we calculated the AAR value for each protein in each data set using the Secret-AAR web-server (http://microbiomics.ibt.unam.mx/tools/aar/index.php) and reported the average unless stated otherwise [55]. Then, we used a Mann-Whitney statistical test to establish any significant differences between the AAR values of the different protein data sets.

## Supplementary Information

**Additional file 1: Table S1.** Complete metadata of the 15 clinical isolates of *M. abscessus* genomes sequenced.

**Additional file 2: Figure S1.** Bioinformatics pipeline to indentify and analyze the secreted proteins of *M. abscessus*.

**Additional file 3: Table S2.** Statistic data of the de novo assemblies for the sequenced isolates.

**Additional file 4: Table S3.** Comparison of the core secretome of each subspecies vs NCBI genomes.

**Additional file 5: Table S4.** AAR values for random constructed secretomes of the rough and smooth phenotypes.

**Additional file 6: Figure S2.** GO enrichment analysis of enzymes for *M. abscessus* ATCC19977. Percentage of sequences annotated with each GO term for the secreted proteins (blue) and the complete proteins in the genome (red).

**Additional file 7: Table S5.** List of 222 M. *abscessus *proteins with homologues in *M. tuberculosis* H37Rv and with previous experimental support for secretion according to Cornejo-Granados et al. [19].

**Additional file 8: Table S6.** Potential drug targets for 13 proteins shared between *M. tuberculosis* H37Rv and M. *abscessus* ATCC19977.

## Data Availability

The reference genomes analyzed for *M. abscessus* ATCC19977 and *M. tuberculosis* H37Rv were taken from NCBI, under GenBank IDs CU458896.1 and NC_000962.3, respectively. The Whole Genome Shotgun project has been deposited at NCBI, under BioProject PRJNA646278. It can be accessed with the link https://www.ncbi.nlm.nih.gov/bioproject/PRJNA646278. All the predicted secretomes were deposited in the Secret-AAR web-server (http://microbiomics.ibt.unam.mx/tools/aar/index.php). Additional data supporting the conclusions of this article are included within the article and its additional file(s).

## Abbreviations

MAB: *Mycobacterium abscessus*
NTM: Non-tuberculous mycobacterium
MTBC: *M. tuberculosis complex*
MTB: *M. tuberculosis*
TB: Tuberculosis
ES: Excreted / secreted
R: Rough
S: Smooth
MAB_A_: *M. abscessus* subsp. *abscessus*
MAB_B_: *M. abscessus* subsp. *bolletii*
MAB_M_: *M. abscessus* subsp. *massiliense*
non-ES: Not-secreted
TM: Transmembrane
incell: Intracellular
AAR: Abundance of Antigenic Regions
MLST: Multilocus Sequence Typing
ST: Sequence Type
