# Supplementary material for: Secretome characterization of clinical isolates from the Mycobacterium abscessus complex provides insight into antigenic differences
Source: BMC Genomics. 2021 May 25;22:385. doi: 10.1186/s12864-021-07670-7 (PMC8152154; doi:10.1186/s12864-021-07670-7)
Supplement: Supplementary file 1 — Additional file 1: Table S1. Complete metadata of the 15 clinical isolates of M. abscessus genomes sequenced. [file 12864_2021_7670_MOESM1_ESM.pdf]

Table S1. Complete metadata of the fifteen clinical isolates of *M. abscessus* genomes sequenced.

**Species**	**Accession number**	**Genome ID**	**Origin**	**Phenotype**	**Total predicted proteins**	**ES proteins**	**non-ES proteins**	**incell proteins**	**TM proteins**	**mean ES AAR**	**mean non-ES AAR**	**mean incell AAR**	**mean TM AAR**
M. abscessus subsp. abscessus	CU458896.1	reference strain ATCC19977	-	-	4,942	886	4,056	3,196	860	40.78	43.72	38.96	61.60
M. abscessus subsp. abscessus	GCA_015499845.1	4549-15	sputum	rough	5,105	929	4,176	3,213	963	40.66	43.48	39.07	58.34
	GCA_015499865.1	11351-15	sputum	rough	5,138	966	4,172	3,215	957	40.12	43.63	39.05	59.20
	GCA_015499835.1	8844-15	skin	smooth	4,854	956	3,898	2,974	924	39.81	43.87	39.04	59.58
	GCA_015499805.1	3563-15	sputum	smooth	5,239	968	4,271	3,271	1,000	40.11	43.43	38.99	58.15
	GCA_015499795.1	12389-15	sputum	smooth	5,276	990	4,286	3,314	972	40.19	43.30	38.95	58.35
	GCA_015499765.1	2677-16	sputum	smooth	4,900	919	3,981	3,024	957	40.68	43.87	39.06	59.28
	GCA_015499745.1	2572-17	tissue (breast implant)	NA	4,847	874	3,973	3,039	934	40.47	43.76	38.98	59.58
M. abscessus subsp. massiliense	GCA_015499715.1	14479-15	sputum	rough	5,120	962	4,158	3,190	968	40.89	43.65	38.85	59.64
	GCA_015499735.1	10896-16	sputum	rough	5,109	950	4,159	3,192	967	40.86	43.54	38.86	59.15
	GCA_015499695.1	10003-15	sputum	smooth	4,835	891	3,944	3,017	927	41.01	43.61	38.86	59.30
	GCA_015499655.1	16155-15	sputum	smooth	4,884	898	3,986	3,061	925	40.84	43.50	38.83	59.14
M. abscessus subsp. bolletii	GCA_015499665.1	11702-16	sputum	rough	5,079	931	4,148	3,177	971	40.22	43.56	38.90	58.97
	GCA_015499625.1	713-16	lymph node	rough	5,456	1,037	4,419	3,401	1,018	40.78	43.42	38.95	58.57
	GCA_015499615.1	7742-15	blood culture	smooth	4,913	885	4,028	3,067	961	41.69	43.69	39.04	58.77
	GCA_015499585.1	13116-16	lymph node	smooth	5,305	990	4,315	3,312	1,003	40.44	43.46	38.82	58.96
