# Supplementary material for: Secretome characterization of clinical isolates from the Mycobacterium abscessus complex provides insight into antigenic differences
Source: BMC Genomics. 2021 May 25;22:385. doi: 10.1186/s12864-021-07670-7 (PMC8152154; doi:10.1186/s12864-021-07670-7)
Supplement: Supplementary file 2 — Additional file 2: Figure S1. Bioinformatics pipeline to indentify and analyze the secreted proteins of M. abscessus. [file 12864_2021_7670_MOESM2_ESM.pdf]

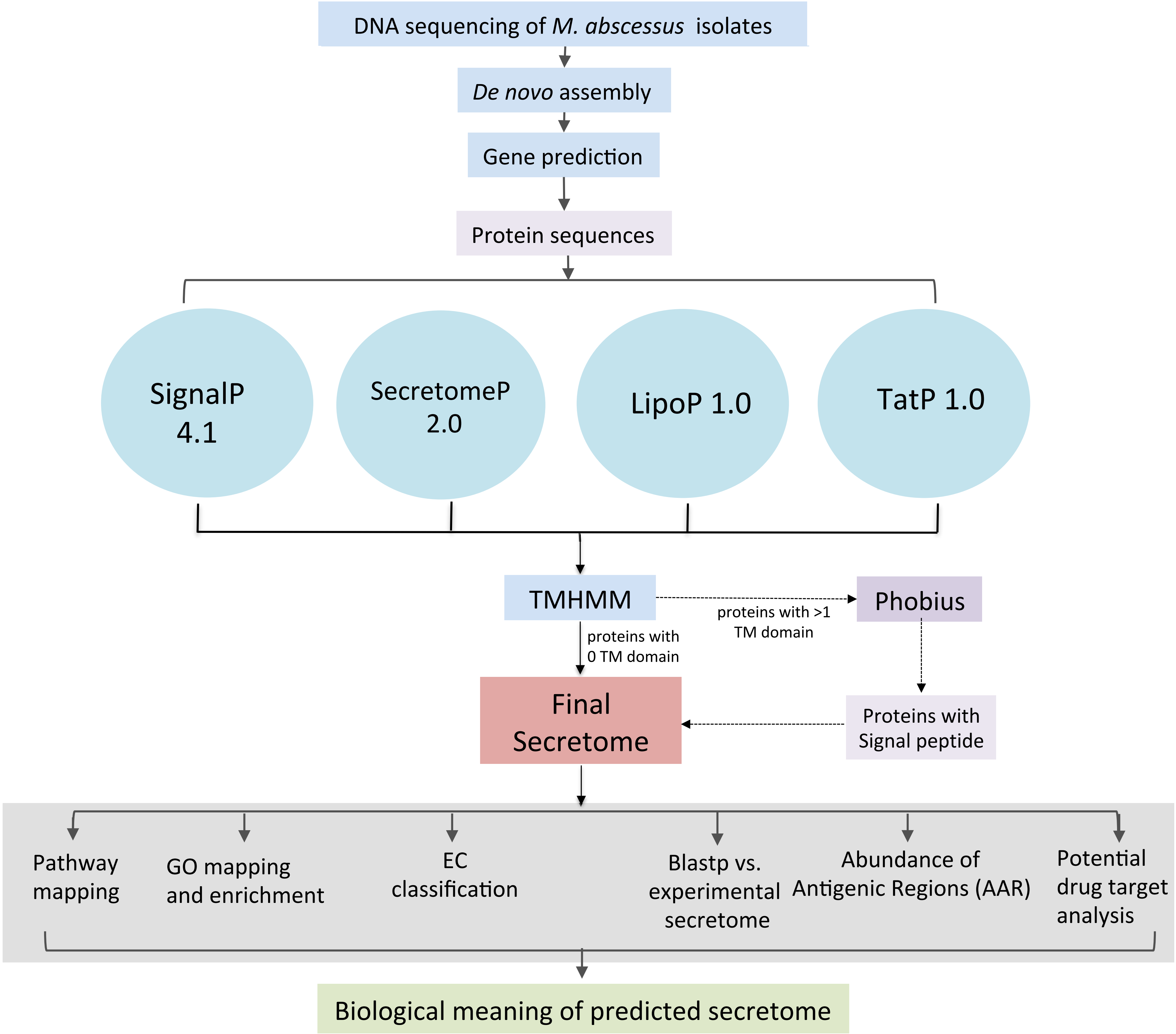

Supplementary Figure S1. Bioinformatics pipeline to identify and analyze the secreted proteins of *M. abscessus*.
