# Supplementary material for: Secretome characterization of clinical isolates from the Mycobacterium abscessus complex provides insight into antigenic differences
Source: BMC Genomics. 2021 May 25;22:385. doi: 10.1186/s12864-021-07670-7 (PMC8152154; doi:10.1186/s12864-021-07670-7)
Supplement: Supplementary file 3 — Additional file 3: Table S2. Statistic data of the de novo assemblies for the sequenced isolates. [file 12864_2021_7670_MOESM3_ESM.pdf]

Table S2.| Statistic data of the de novo assemblies for the sequenced isolates.

Genome	Strain	Total reads	N50	Mean base coverage	Largest Contig	Total Contigs
4549-15	*M. abscessus*	1,841,762	241,795	217.25	479,260	61
11351-15	*M. abscessus*	2,405,256	200,998	284.07	455,684	75
8844-15	*M. abscessus*	2,483,126	220,207	307.33	366,033	60
3563-15	*M. abscessus*	2,941,828	218,353	343.09	775,036	52
12389-15	*M. abscessus*	2,241,946	232,848	257.56	357,818	63
2677-16	*M. abscessus*	2,755,836	347,263	337.93	701,340	71
2572-17	*M. abscessus*	2,576,134	337,055	317.67	1,047,246	38
11702-16	*M. bolletii*	2,255,592	652,527	268.14	1,269,368	53
713-16	*M. bolletii*	2,641,622	384,872	296.01	607,861	68
7742-15	*M. bolletii*	2,989,318	302,394	365.44	962,231	47
13116-16	*M. bolletii*	2,785,738	344,725	319.87	1,196,314	55
14479-15	*M. massiliense*	3,100,556	327,410	368.37	722,347	64
10896-16	*M. massiliense*	2,510,338	327,557	298.78	522,805	47
10003-15	*M. massiliense*	2,667,154	511,575	329.83	1,346,120	39
16155-15	*M. massiliense*	2,825,456	339,641	345.85	1,087,576	78
