# Supplementary material for: Secretome characterization of clinical isolates from the Mycobacterium abscessus complex provides insight into antigenic differences
Source: BMC Genomics. 2021 May 25;22:385. doi: 10.1186/s12864-021-07670-7 (PMC8152154; doi:10.1186/s12864-021-07670-7)
Supplement: Supplementary file 4 — Additional file 4: Table S3. Comparison of the core secretome of each subspecies vs NCBI genomes. [file 12864_2021_7670_MOESM4_ESM.pdf]

Supplementary Table S3 | Comparisson of the core secretome of each subspecies vs NCBI genomes.

	GenBank Accession number	Numer of ES core proteins present in the new genomes	% of ES core proteins present in the new genomes	Average %
Comparisson to M. abscessus sbsp. *abscessus genomes (735 core ES proteins)*	GCA_900141265.1	734	99.86	99.78
	GCA_900140885.1	733	99.73	
	GCA_900140835.1	733	99.73	
	GCA_900140825.1	732	99.59	
	GCA_900140545.1	732	99.59	
	GCA_900140475.1	733	99.73	
	GCA_900140045.1	734	99.86	
	GCA_900140005.1	734	99.86	
	GCA_900139935.1	732	99.59	
	GCA_900139505.1	734	99.86	
	GCA_900137175.1	733	99.73	
	GCA_900137165.1	734	99.86	
	GCA_900137155.1	734	99.86	
	GCA_900137145.1	734	99.86	
	GCA_900136765.1	734	99.86	
	GCA_900136595.1	734	99.86	
	GCA_900135095.1	733	99.73	
	GCA_900135045.1	734	99.86	
	GCA_900132315.1	733	99.73	
	GCA_900132295.1	734	99.86	
Comparisson to M. abscessus sbsp. bolletii genomes (794 core ES proteins)	GCA_900132985.1	778	97.98	99.12
	GCA_900133045.1	790	99.50	
	GCA_900133055.1	788	99.24	
	GCA_900133105.1	788	99.24	
	GCA_900133625.1	788	99.24	
	GCA_900133635.1	788	99.24	
	GCA_900133785.1	787	99.12	
	GCA_900134275.1	785	98.87	
	GCA_900134285.1	784	98.74	
	GCA_900134335.1	787	99.12	
	GCA_900134535.1	787	99.12	
	GCA_900135035.1	790	99.50	
	GCA_900136205.1	792	99.75	
	GCA_900136235.1	792	99.75	
	GCA_900136655.1	789	99.37	
	GCA_900137535.1	789	99.37	
	GCA_900137885.1	786	98.99	
	GCA_900139995.1	781	98.36	
	GCA_900141605.1	784	98.74	
	GCF_900131565.1	787	99.12	
Comparisson to M. abscessus sbsp. massiliense genomes (813 core ES proteins)	GCA_900130325.1	805	99.02	98.59
	GCA_900130675.1	802	98.65	
	GCA_900130855.1	804	98.89	
	GCA_900131585.1	798	98.15	
	GCA_900134075.1	803	98.77	
	GCA_900135905.1	804	98.89	
	GCA_900135915.1	802	98.65	
	GCA_900137185.1	803	98.77	
	GCA_900138505.1	802	98.65	
	GCA_900138535.1	802	98.65	
	GCA_900138705.1	806	99.14	
	GCA_900138715.1	806	99.14	
	GCA_900139775.1	801	98.52	
	GCA_900139915.1	797	98.03	
	GCA_900140635.1	795	97.79	
	GCA_900140755.1	795	97.79	
	GCA_900141015.1	800	98.40	
	GCA_900141505.1	801	98.52	
	GCA_900141515.1	803	98.77	
	GCA_900169205.1	802	98.65	
