# Supplementary material for: Secretome characterization of clinical isolates from the Mycobacterium abscessus complex provides insight into antigenic differences
Source: BMC Genomics. 2021 May 25;22:385. doi: 10.1186/s12864-021-07670-7 (PMC8152154; doi:10.1186/s12864-021-07670-7)
Supplement: Supplementary file 5 — Additional file 5: Table S4. AAR values for random constructed secretomes of the rough and smooth phenotypes. [file 12864_2021_7670_MOESM5_ESM.pdf]

Table S4.| AAR values for random constructed secretomes of the rough and smooth phenotypes.

	Number of proteins in the set	empirical *p* value	
M. abs sbsp. *massiliense* unique rough proteins	109	0.01	sets with significative *p* value <0.05
M. abs sbsp. *bolletii* unique rough proteins	48	0.04	
M. abs sbsp. *abscessus* unique smooth proteins	9	0.19	
M. abs sbsp. *bolletii* unique smooth proteins	35	0.37	
M. abs sbsp. *abscessus* unique rough proteins	93	0.58	
M. abs sbsp. *massiliense* unique smooth proteins	76	0.93	
