# Supplementary material for: Secretome characterization of clinical isolates from the Mycobacterium abscessus complex provides insight into antigenic differences
Source: BMC Genomics. 2021 May 25;22:385. doi: 10.1186/s12864-021-07670-7 (PMC8152154; doi:10.1186/s12864-021-07670-7)
Supplement: Supplementary file 6 — Additional file 6: Figure S2. GO enrichment analysis of enzymes for M. abscessus ATCC19977. Percentage of sequences annotated with each GO term for the secreted proteins (blue) and the complete proteins in the genome (red). [file 12864_2021_7670_MOESM6_ESM.pdf]

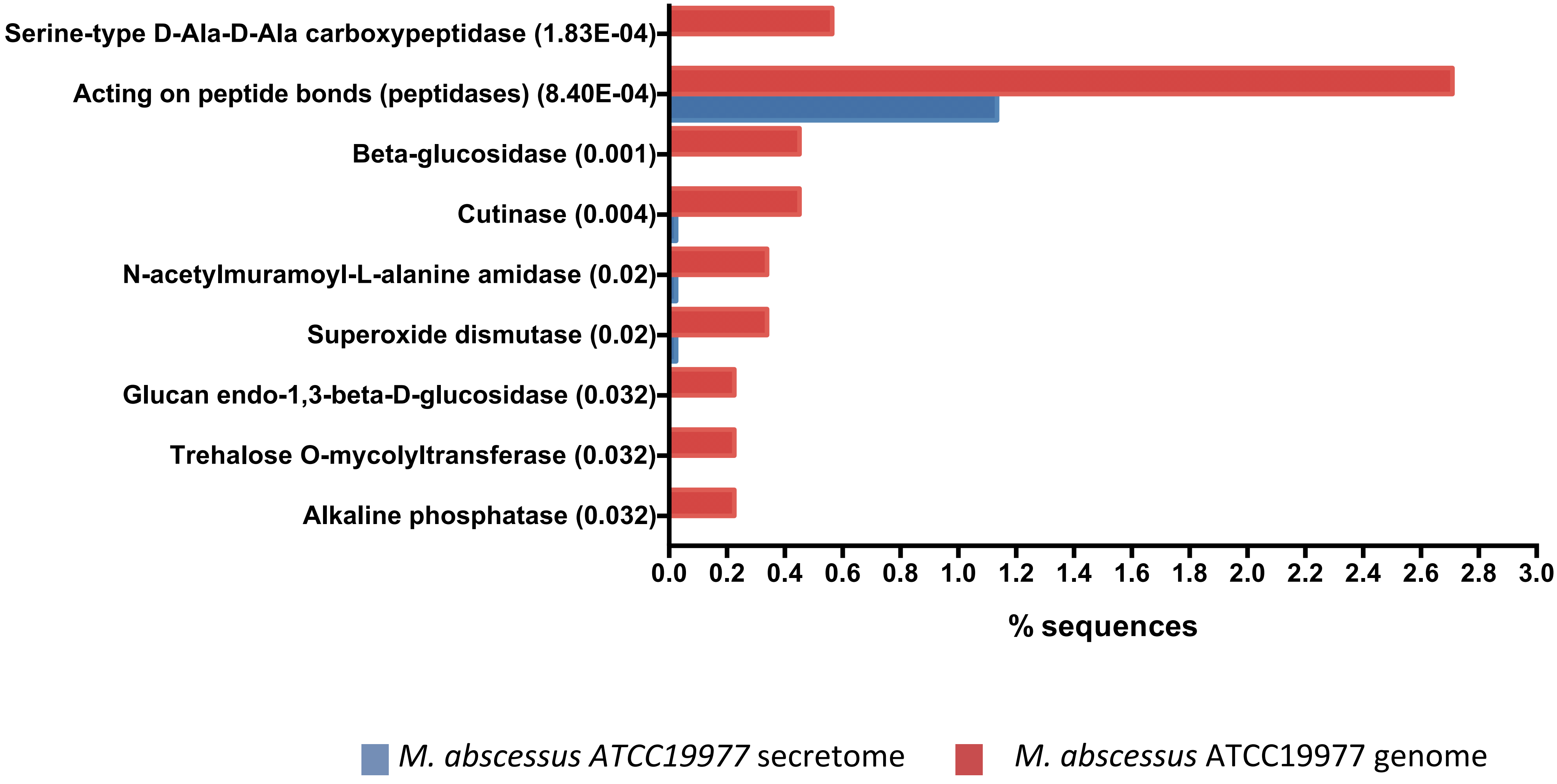

Supplementary Fig S2. GO enrichment analysis of Enzymes for the *M. abscessus* ATCC19977. Percentage of sequences annotated with each GO term for the secretome proteins (blue) and the complete proteins in the genome (red).
