# Supplementary material for: Secretome characterization of clinical isolates from the Mycobacterium abscessus complex provides insight into antigenic differences
Source: BMC Genomics. 2021 May 25;22:385. doi: 10.1186/s12864-021-07670-7 (PMC8152154; doi:10.1186/s12864-021-07670-7)
Supplement: Supplementary file 7 — Additional file 7: Table S5. List of 222 M. abscessus proteins with homologues in M. tuberculosis H37Rv and with previous experimental support for secretion according to Cornejo-Granados et al. [19]. [file 12864_2021_7670_MOESM7_ESM.pdf]

Supplementary Table S5 | List of 222 *M. abscessus* proteins with homologues in *M. tuberculosis* H37Rv and with previous experimental support for secretion according to [Cornejo-Granados et al., 2017 paper]".

	Protein name	Description
1	YP_001700711.1	Conserved hypothetical protein (plasmid)
2	YP_001700767.1	Hypothetical protein MAB_0010c
3	YP_001700781.1	Peptidyl-prolyl cis-trans isomerase
4	YP_001700795.1	Conserved hypothetical protein
5	YP_001700847.1	Conserved hypothetical protein
6	YP_001700862.1	Hypothetical protein MAB_0108c
7	YP_001700866.1	Hypothetical protein MAB_0112
8	YP_001700902.1	PPE family protein
9	YP_001700905.1	Conserved hypothetical protein
10	YP_001700922.1	Putative N-acetymuramoyl-L-alanine amidase
11	YP_001700923.1	Putative exported repetitive protein precursor
12	YP_001701031.1	Probable amino acid ABC transporter, permease
13	YP_001701057.1	Hypothetical protein MAB_0304
14	YP_001701074.1	Hypothetical protein MAB_0321c
15	YP_001701078.1	Hypothetical protein MAB_0325c
16	YP_001701111.1	Hypothetical conserved membrane protein PknM or conserved lipoprotein LppH
17	YP_001701113.1	Conserved hypothetical protein
18	YP_001701114.1	Conserved hypothetical protein
19	YP_001701121.1	Short-chain dehydrogenase/reductas
20	YP_001701129.1	Conserved hypothetical protein (peptidase?)
21	YP_001701177.1	Putative protease
22	YP_001701206.1	Conserved hypothetical protein
23	YP_001701209.1	Hypothetical protein MAB_0456
24	YP_001701213.1	Hypothetical protein MAB_0460
25	YP_001701267.1	Probable oxidoreductase EphD
26	YP_001701292.1	Conserved hypothetical transmembrane protein
27	YP_001701361.1	Conserved hypothetical protein
28	YP_001701380.1	Conserved hypothetical protein
29	YP_001701382.1	Conserved hypothetical protein
30	YP_001701410.1	Conserved hypothetical protein
31	YP_001701416.1	PE family protein
32	YP_001701434.1	Hypothetical conserved membrane protein
33	YP_001701435.1	Hypothetical conserved protein
34	YP_001701467.1	Probable dehydrogenase/reductase
35	YP_001701472.1	Putative oligopeptide ABC transporter, ATP-binding protein
36	YP_001701473.1	Putative triacylglycerol lipase precursor
37	YP_001701484.1	Conserved hypothetical protein
38	YP_001701485.1	Hypothetical protein MAB_0735
39	YP_001701486.1	Hypothetical protein MAB_0736
40	YP_001701490.1	Probable thiosulfate sulfurtransferase (CysA)
41	YP_001701496.1	Putative phosphate ABC transporter, phosphate-binding protein
42	YP_001701525.1	Hypothetical protein MAB_0775
43	YP_001701528.1	Conserved hypothetical protein (lipoprotein LppU?)
44	YP_001701579.1	Conserved hypothetical protein
45	YP_001701589.1	Hypothetical protein MAB_0841
46	YP_001701617.1	Probable resuscitation-promoting factor RpfA
47	YP_001701632.1	Hypothetical protein MAB_0884c
48	YP_001701633.1	Hypothetical lipoprotein lpqH precursor
49	YP_001701713.1	Hypothetical protein MAB_0967
50	YP_001701720.1	Conserved hypothetical protein
51	YP_001701724.1	Hypothetical protein MAB_0978
52	YP_001701750.1	Putative MCE family protein
53	YP_001701753.1	Putative MCE family protein
54	YP_001701754.1	Putative MCE family protein
55	YP_001701757.1	Hypothetical protein MAB_1013
56	YP_001701770.1	Conserved hypothetical protein
57	YP_001701857.1	Hypothetical protein MAB_1115
58	YP_001701872.1	Conserved hypothetical protein
59	YP_001701906.1	Putative conserved lipoprotein LpqU
60	YP_001701920.1	Conserved hypothetical protein
61	YP_001701935.1	Conserved hypothetical protein (lipolytic enzyme G-D-S-L?)
62	YP_001701943.1	Hypothetical transcription elongation factor GreA
63	YP_001702022.1	Hypothetical protein MAB_1280c
64	YP_001702045.1	Hypothetical protein MAB_1303c
65	YP_001702056.1	Hypothetical protein MAB_1314
66	YP_001702057.1	Putative lipoprotein LpqW
67	YP_001702064.1	Hypothetical protein MAB_1322
68	YP_001702065.1	Hypothetical protein MAB_1323
69	YP_001702085.1	Conserved hypothetical protein (nuclease?)
70	YP_001702107.1	Conserved hypothetical protein
71	YP_001702142.1	Putative lipoprotein LprE precursor
72	YP_001702156.1	Putative lipoprotein LprB precursor
73	YP_001702157.1	Putative lipoprotein LprC precursor
74	YP_001702177.1	Probable homoserine kinase (ThrB)
75	YP_001702180.1	Hypothetical protein MAB_1440c
76	YP_001702205.1	Conserved hypothetical protein thioredoxin-like
77	YP_001702206.1	Possible lipoprotein peptidase LpqM
78	YP_001702210.1	Possible lipoprotein peptidase LpqM
79	YP_001702247.1	Conserved hypothetical protein
80	YP_001702251.1	Conserved hypothetical protein
81	YP_001702266.1	Putative short chain dehydrogenase/reductase
82	YP_001702270.1	Probable conserved lipoprotein LppS
83	YP_001702282.1	Conserved hypothetical protein
84	YP_001702316.1	Conserved hypothetical protein
85	YP_001702336.1	Conserved hypothetical protein

86	YP_001702437.1	Putative Mce family protein
87	YP_001702438.1	Putative Mce family protein
88	YP_001702439.1	Putative Mce family protein
89	YP_001702445.1	Conserved hypothetical protein
90	YP_001702539.1	Bacteriophage protein
91	YP_001702541.1	Bacteriophage protein
92	YP_001702567.1	Hypothetical protein MAB_1830
93	YP_001702569.1	Conserved hypothetical protein
94	YP_001702570.1	Conserved hypothetical protein
95	YP_001702572.1	Immunogenic protein MPT64 precursor
96	YP_001702577.1	Putative beta-glucanase
97	YP_001702628.1	Conserved hypothetical protein
98	YP_001702632.1	Conserved hypothetical protein
99	YP_001702710.1	Putative secreted protein
100	YP_001702717.1	Conserved hypothetical protein
101	YP_001702838.1	Conserved hypothetical protein
102	YP_001702895.1	Putative lipoprotein LppK precursor
103	YP_001702896.1	Hypothetical low molecular weight antigen Mtb12
104	YP_001703063.1	Conserved hypothetical protein
105	YP_001703114.1	Hypothetical lipoprotein LpqH precursor
106	YP_001703138.1	Conserved hypothetical protein
107	YP_001703155.1	Conserved hypothetical protein
108	YP_001703156.1	Conserved hypothetical protein
109	YP_001703168.1	Molybdenum ABC transporter ModA, periplasmic
110	YP_001703171.1	Conserved hypothetical protein (fibronectin-attachment?)
111	YP_001703191.1	Conserved hypothetical protein
112	YP_001703209.1	Conserved hypothetical protein
113	YP_001703223.1	Conserved hypothetical protein (9 kDa antigen)
114	YP_001703235.1	Hypothetical protein MAB_2500
115	YP_001703267.1	Hypothetical protein MAB_2532
116	YP_001703294.1	Probable peptidyl-prolyl cis-trans isomerase
117	YP_001703315.1	Hypothetical protein MAB_2580c
118	YP_001703426.1	Conserved hypothetical protein
119	YP_001703431.1	Hypothetical protein MAB_2697c
120	YP_001703460.1	Hypothetical invasion protein Inv2
121	YP_001703461.1	Hypothetical invasion protein Inv1
122	YP_001703472.1	Probable thioredoxin TrxB
123	YP_001703474.1	Conserved hypothetical protein
124	YP_001703532.1	Conserved hypothetical protein
125	YP_001703533.1	Conserved hypothetical protein
126	YP_001703534.1	Conserved hypothetical protein
127	YP_001703538.1	Conserved hypothetical protein
128	YP_001703539.1	Lipoprotein LprG precursor (27 kDa lipoprotein)
129	YP_001703585.1	Hypothetical protein MAB_2852c
130	YP_001703608.1	Beta-lactamase precursor (Penicillinase)
131	YP_001703611.1	Conserved hypothetical protein
132	YP_001703677.1	Putative Mce family protein
133	YP_001703703.1	Putative oxidoreductase
134	YP_001703704.1	Conserved hypothetical protein
135	YP_001703713.1	Putative lipoprotein LppU
136	YP_001703741.1	Probable RNA polymerase sigma factor RpoD (Sigma-A)
137	YP_001703763.1	Conserved hypothetical protein
138	YP_001703783.1	Putative glutamate ABC transporter, periplasmic protein
139	YP_001703834.1	Putative short chain dehydrogenase/reductase
140	YP_001703883.1	Hypothetical protein MAB_3152c
141	YP_001703896.1	Conserved hypothetical protein
142	YP_001703912.1	Probable lipoprotein LppI
143	YP_001703950.1	Hypothetical protein MAB_3220
144	YP_001703991.1	Probable lipoprotein LpqH precursor
145	YP_001703994.1	Hypothetical protein MAB_3264c
146	YP_001704077.1	Conserved hypothetical protein
147	YP_001704078.1	Conserved hypothetical protein
148	YP_001704079.1	Conserved hypothetical protein
149	YP_001704120.1	Probable FeIII-dicitrate-binding periplasmic lipoprotein
150	YP_001704184.1	Conserved hypothetical protein
151	YP_001704212.1	NADPH-ferredoxin reductase FprA
152	YP_001704214.1	Conserved hypothetical protein
153	YP_001704392.1	Conserved hypothetical protein
154	YP_001704455.1	Hypothetical protein MAB_3727c
155	YP_001704460.1	10 kDa chaperonin (GroES)
156	YP_001704474.1	Conserved hypothetical protein
157	YP_001704481.1	Conserved hypothetical protein
158	YP_001704491.1	Probable cutinase cut2 precursor
159	YP_001704493.1	Probable cutinase cut3 precursor
160	YP_001704494.1	Probable cutinase cut3 precursor
161	YP_001704528.1	Conserved hypothetical protein
162	YP_001704538.1	Probable cutinase Cut4
163	YP_001704581.1	Conserved hypothetical protein
164	YP_001704582.1	Conserved hypothetical protein
165	YP_001704583.1	Putative lipoprotein LprC
166	YP_001704584.1	Putative lipoprotein LprB precursor
167	YP_001704644.1	Probable conserved secreted protein
168	YP_001704647.1	Putative short chain dehydrogenase/reductase
169	YP_001704700.1	Conserved hypothetical protein
170	YP_001704704.1	Possible thioredoxin
171	YP_001704759.1	Putative Mce family protein
172	YP_001704761.1	Putative Mce family protein
173	YP_001704762.1	Putative Mce family protein
174	YP_001704763.1	Putative Mce family protein

175	YP_001704781.1	Putative short chain dehydrogenase/reductase
176	YP_001704789.1	Conserved hypothetical protein
177	YP_001704801.1	Lipoprotein LpqH precursor
178	YP_001704807.1	Conserved hypothetical protein
179	YP_001704841.1	Conserved hypothetical protein
180	YP_001704876.1	Hypothetical MCE-family protein LprN
181	YP_001704911.1	Superoxide dismutase
182	YP_001704950.1	Probable glutamine-binding protein GlnH
183	YP_001704963.1	Putative amino acid ABC transporter, substrate-binding protein
184	YP_001705003.1	Probable conserved lipoprotein DsbF
185	YP_001705011.1	Hypothetical protein MAB_4284c
186	YP_001705017.1	Conserved hypothetical protein
187	YP_001705018.1	Conserved hypothetical protein
188	YP_001705019.1	Putative transcriptional regulator, LuxR family
189	YP_001705025.1	Conserved hypothetical protein
190	YP_001705026.1	Conserved hypothetical protein
191	YP_001705043.1	Conserved hypothetical protein
192	YP_001705051.1	Hypothetical protein MAB_4325c
193	YP_001705115.1	Putative ABC transporter, periplasmic substrate-binding
194	YP_001705128.1	Hypothetical protein MAB_4404
195	YP_001705129.1	Putative serine esterase, cutinase family
196	YP_001705181.1	Putative secreted hydrolase
197	YP_001705235.1	Putative Mce family protein
198	YP_001705236.1	Putative Mce family protein
199	YP_001705238.1	Putative Mce family protein
200	YP_001705251.1	Conserved hypothetical protein
201	YP_001705260.1	Conserved hypothetical protein
202	YP_001705287.1	Putative Mce family protein
203	YP_001705290.1	Putative Mce family protein
204	YP_001705318.1	Putative Mce family protein
205	YP_001705321.1	Putative Mce family protein
206	YP_001705329.1	Conserved hypothetical protein
207	YP_001705350.1	Hypothetical protein MAB_4627
208	YP_001705424.1	Conserved hypothetical protein
209	YP_001705462.1	Possible beta-1,3-glucanase
210	YP_001705496.1	Hypothetical protein MAB_4774c
211	YP_001705499.1	Possible cellulase CelA (endoglucanase)
212	YP_001705504.1	Conserved hypothetical protein
213	YP_001705523.1	Possible twin-arginine translocation pathway
214	YP_001705574.1	Phosphate ABC transporter, periplasmic protein
215	YP_001705620.1	Single-stranded DNA-binding protein
216	YP_001705625.1	Hypothetical protein MAB_4903
217	YP_001705636.1	Hypothetical protein MAB_4914c
218	YP_001705644.1	Putative short-chain dehydrogenase/reductase
219	YP_001705646.1	Conserved hypothetical protein
220	YP_001705647.1	Hypothetical protein MAB_4925
221	YP_001705657.1	MutT/NUDIX family protein
222	YP_001705663.1	Thioredoxin (Trx)
