# Supplementary material for: Secretome characterization of clinical isolates from the Mycobacterium abscessus complex provides insight into antigenic differences
Source: BMC Genomics. 2021 May 25;22:385. doi: 10.1186/s12864-021-07670-7 (PMC8152154; doi:10.1186/s12864-021-07670-7)
Supplement: Supplementary file 8 — Additional file 8: Table S6. Potential drug targets for 13 proteins shared between M. tuberculosis H37Rv and M. abscessus ATCC19977. [file 12864_2021_7670_MOESM8_ESM.pdf]

Supplementary Table S6. Potential drug targets for 13 proteins shared between *M. tuberculosis H37Rv* and *M. abscessus* ATCC19977.

Protein	Target	E value	Approved drug
YP _001700781.1	Peptidyl-prolyl cis-trans isomerase	1.998E-21	Cyclosporine, copper
YP_001700922.1	Putative N-acetymuramoyl-L-alanine amidase	1.450E-11	Copper, zinc, zinc acetate, zinc chloride, zinc sulfate
YP_001701267.1	Probable oxidoreductase EphD	4.398E-18	NADH
YP_001701361.1	Conserved hypothetical protein	2.168E-52	Delamanid, pretomanid
YP_001701467.1	Probable dehydrogenase/reductase	3.155E-11	Vitamin A
YP_001701490.1	Probable thiosulfate sulfurtransferase (CysA)	6.917E-28	Thiosulfuric acid
YP_001702539.1	Bacteriophage protein	4.490E-29	Cefalotin, Formaldehyde
YP_001703741.1	Probable RNA polymerase sigma factor RpoD (Sigma-A)	6.565E-114	Fidaxomicin
YP_001703834.1	Putative short chain dehydrogenase/reductase	5.959E-14	Vitamin A
YP_001704212.1	NADPH-ferredoxin reductase FprA	2.671E-172	Flavin adenine dinucleotide
YP_001704911.1	Superoxide dismutase [Cu-Zn] precursor	2.891E-07	Vitamin E, Cisplatin, Carboplatin, Oxaliplatin, Copper, Zinc
YP_001705644.1	Putative short-chain dehydrogenase/reductase	3.002E-16	Vitamin A
YP_001705663.1	Thioredoxin (Trx)	4.598E-13	Copper, zinc, zinc acetate, zinc chloride, zinc sulfate
